# T cell mediated hypersensitivity to previously tolerated iodinated contrast media precipitated by introduction of atezolizumab

**DOI:** 10.1136/jitc-2021-002521

**Published:** 2021-05-28

**Authors:** Sean Hammond, Anna Olsson-Brown, Joshua Gardner, Paul Thomson, Serat-E Ali, Carol Jolly, Dan Carr, Lorenzo Ressel, Munir Pirmohamed, Dean Naisbitt

**Affiliations:** 1 Department of Molecular and Clinical Pharmacology, University of Liverpool, Liverpool, UK; 2 ApconiX, Alderley Park, Alderley Edge, UK; 3 Department of Veterinary Pathology and Public Health, Leahurst Campus, Neston, UK

**Keywords:** adaptive immunity, lymphocyte activation, T-lymphocytes

## Abstract

Many adverse reactions associated with immune checkpoint inhibitor (ICI) treatments are immunologically driven and may necessitate discontinuation of the ICI. Herein, we present a patient who had been administered the radio contrast media amidotrizoate multiple times without issue but who then developed a Stevens-Johnson syndrome reaction after coadministration of atezolizumab. Causality was confirmed by a positive re-challenge with amidotrizoate and laboratory investigations that implicated T cells. Importantly, the introduction of atezolizumab appears to have altered the immunologic response to amidotrizoate in terms of the tolerance–elicitation continuum. Proof of concept studies demonstrated enhancement of recall responses to a surrogate antigen panel following *in-vitro* (healthy donors) and *in-vivo* (ICI patients) administrations of ICIs. Our findings highlight the importance of considering all concomitant medications in patients on ICIs who develop immune-mediated adverse reactions. In the event of some immune-related adverse reactions, it may be critical to identify the culprit antigen-forming entity that the ICIs have altered the perception of rather than simply attribute causality to the ICI itself in order to optimize both patient safety and treatment of malignancies.

## Introduction

The emergence of immune checkpoint inhibitors (ICIs) as a therapeutic option has dramatically altered the oncologic treatment landscape. Impressive efficacy data have been accruing over the last few years with the use of antibodies targeted at the PD1 and CTLA-4 coinhibitory pathways. Unfortunately, the use of checkpoint inhibitors has also been associated with a neoteric spectrum of adverse reactions, coined immune-related adverse events (IrAEs). The mechanism thought to underlie the IrAEs is ICI-mediated perturbation of self and xenobiotic related antigens. ICI mediated aberration of T cell responses to a number of compounds was first reported in various experimental models incorporating blockade/ablation of the CTLA-4 and PD pathways.^1 2^ This phenomenon has now been observed clinically, with hypersensitivity reactions to a number of pharmaceutical compounds shown to be exacerbated by the use of ICIs,^3 4^ sometimes with fatal consequences.^5^

We report the case of a patient who developed Stevens-Johnson syndrome (SJS) to a previously tolerated iodinated contrast medium, amidotrizoate, after the use of atezolizumab, a PD-L1 blocker. We also present supporting *in-vitro* evidence that provides novel mechanistic insight.

## Methods

The patient was recruited from the Clatterbridge Cancer Centre, Liverpool, UK, at the onset of the initial severe skin reaction considered to be due to the ICI agent. Subsequent sampling occurred at the onset of the second severe episode. Clinical data and photographic images were recorded and anonymized. Biologic samples (peripheral blood mononuclear cells (PBMCs) and skin biopsies) were obtained in a longitudinal manner periodically during both the initial and recurrent episodes. We also obtained blood samples from seven other patients prior to and 1 week following initiation of treatment with ICIs.

Bulk PBMC enrichment, limiting dilution, mitogen-driven expansion and initial specificity testing (for amidotrizoate) of monoclonal T cell populations was conducted as described by Gibson *et al*.^2^ An Epstein-Barr virus (EBV) transformed B cell line was generated from the patient’s PBMCs via transformation with supernatant from the EBV-producing cell line, B95.8, serving thereafter as an immortalized autologous source of antigen-presenting cells. Dose-response experiments were undertaken for specific T cell clones (TCC) over a range of amidotrizoate concentrations (100 nM–15 mM). Cytokine release (interferon gamma (IFNγ), granzyme B and perforin) from TCC on challenge with amidotrizoate was evaluated via ELISpot in accordance with manufacturer’s instructions (MabTech, Nacka Strand, Sweden).

Lymphocyte transformation tests (LTT) were conducted as outlined by Pichler and Tilch^6^ with PBMC (1.5×10^5^ cells/well) from healthy donors (n=9) treated with checkpoint blockade antibodies *in-vitro* (anti-PDL-1 5 µg/mL IgG2b mAb, clone 29E.2A3 Biolegend UK, anti-CTLA-4 5 µg/mL IgG1 mAb, clone L3D10, Biolegend UK, or both) and PBMC samples taken from clinical visits of oncology patients (n=7; ipilimumab and nivolumab (n=3); pembrolizumab (n=3); nivolumab (n=1)) prior to and 1 week following initiation of treatment with ICIs to investigate recall responses to an antigen panel (Bandrowski’s base 0.5–5 µM, tetanus toxoid 0.5–5 µg/mL, purified protein derivative 1–5 µg/mL). Media control wells served as baseline controls, with all antigen specific responses normalized to a stimulation index of media control±blocking antibody in order to account for any blockade-induced baseline shift.

## Results

**Figure 1:**
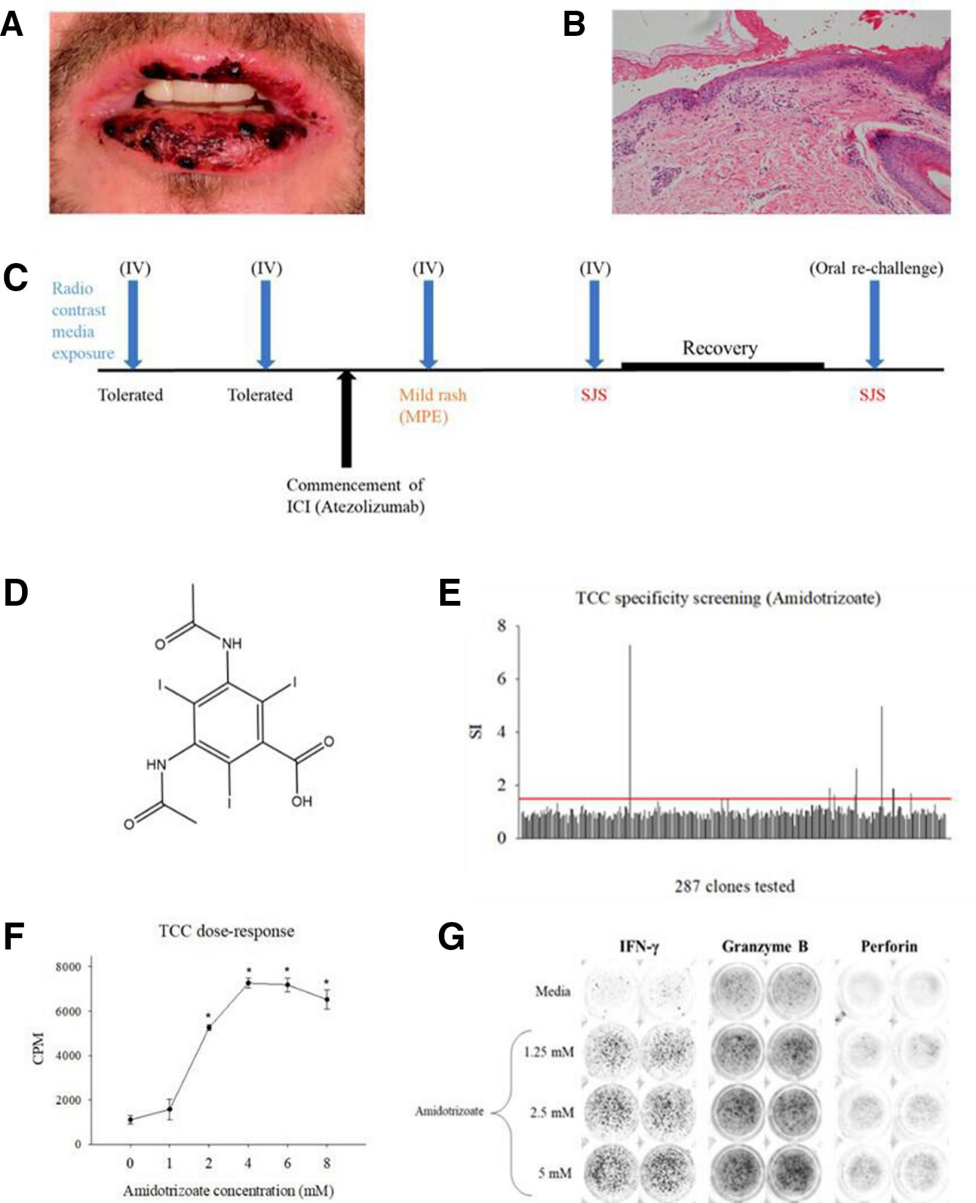
History, clinical presentation and *in-vitro* laboratory work-up of patient. (A) Timeline of patient exposure to amidotrizoate, with introduction of atezolizumab resulting in progressive severe cutaneous adverse reactions and inadvertent confirmation of the causative agent through recrudescence of SJS following recovery. (B) Clinical presentation of blistering and necrotic presentation of oral mucosae consistent with SJS. (C) Histological image depicts full thickness epidermal necrosis and partial dermal detachment from the dermis. (D) Chemical structure of the iodinated radio contrast media amidotrizoate. (E) T cell clone specificity screening. T cell clones (5×10^4^) were cultured with autologous irradiated EBV-transformed B cells (1×10^4^) (96-well U bottomed) and indicated concentrations of amidotrizoate for 48 hours at 37°C, 5% CO_2_. Cultures were then pulsed with [^3^H] thymidine (0.5 µCi/well) and subject to a further 16-hour incubation. Data presented as stimulation indices (SIs) for incorporated radioactivity in drug treated/media controls for duplicate cultures. (F) Dose-proliferation response of amidotrizoate-responsive T cells isolated in B. Data presented as mean±SD counts per minute (CPM), a function of T cell [^3^H] thymidine incorporation with all experimental conditions performed using triplicate cultures. (G) ELISpot well images depicting dose-dependent release of IFNγ, granzyme B, and perforin by T cell clones challenged with amidotrizoate in coculture with autologous EBV-transformed B cells for 48 hours at 37°C, 5% CO_2_. EBV, Epstein-Barr virus; ICI, immune checkpoint inhibitor; SJS, Stevens-Johnson syndrome.

The index patient had been receiving first line ICI treatment with atezolizumab (anti-PD-L1 IgG1 mAb) for the management of metastatic renal cell carcinoma. The patient had previously undergone in excess of 20 contrast-enhanced CT scans. On each occasion, the iodinated contrast agent amidotrizoate was administered intravenously (80 mL at 678 mg/mL; total 54 g) and was well tolerated prior to the recommencement of ICI therapy. He had received 15 cycles of treatment followed by a treatment break to allow for palliative surgery. Following recovery from surgery, the patient recommenced ICI therapy and underwent a CT scan 2 weeks after. Twenty-four hours after exposure to intravenous amidotrizoate, the patient experienced a mild pruritic reaction. Following subsequent exposures, the patient experienced increasingly severe skin manifestations progressing from mild erythema multiforme (EM) to a blistering, desquamating rash consistent with Stevens-Johnson syndrome (SJS). Blistering involved both oral (figure 1B) and genital mucous membranes, widespread targetoid type skin lesions associated with blistering/epidermal loss affecting <10% body surface area. Histology showed full thickness necrosis of the epidermis with partial detachment from the dermis (figure 1C). Eosinophils remained in the normal range. The patient was hospitalized and received 5 days of intravenous methylprednisolone (240 mg (2 mg/kg), daily) with a subsequent protracted corticosteroid wean over a period of 4 months.

The serious cutaneous adverse reaction in this patient was initially attributed to the ICI itself, but the possible role of amidotrizoate could not be excluded. Given the limitation of non-contrast enhanced imaging in malignant disease surveillance, a clinical, risk-informed decision was made to expose the patient to the contrast agent. Thus, 6 months after his previous CT scan, he was rechallenged with oral amidotrizoate (16 mL at 760 mg/mL; 12 g). This culminated in the recrudescence of SJS with the same clinical phenotype within 2 hours of administration (figure 1A). This was initially treated with 10 mg Chlorpheniramine and 200 mg intravenous hydrocortisone, and subsequently with intravenous methylprednisolone (240 mg (2 mg/kg) daily) for 3 days followed by a weaning dose of oral prednisolone. A tumour necrosis factor alpha (TNF-α) inhibitor was not used because of a perceived risk of liver injury. The rapid recurrence of SJS following rechallenge with amidotrizoate provided good evidence that the contrast agent was responsible for the serious skin reactions in this patient. The patient discontinued ICI treatment and has since undergone non-contrast enhanced CT scans. No further episodes of skin reactions have occurred following these alterations. He has experienced an ongoing complete response to treatment.

**Figure 2:**
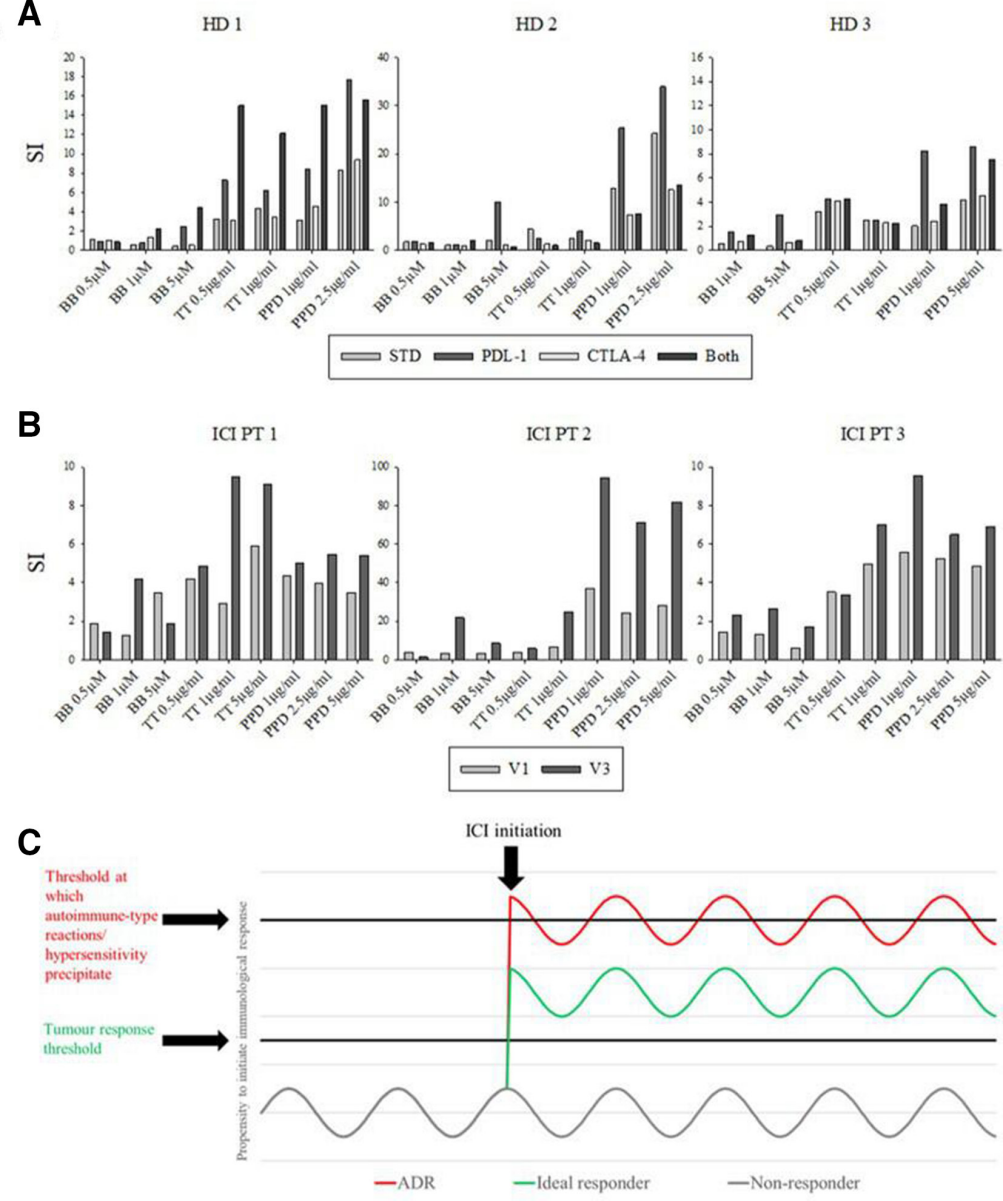
Assessment of ICI-mediated perturbation of recall responses. (A) *In-vitro* administration of ICI blocking antibodies yields enhanced responses to a panel of antigens. (B) LTTs conducted prior to (V1: clinical visit one) and 1 week after (V3: clinical visit 3) *in-vivo* administration of ICI blocking antibodies yields enhanced responses to the antigen panel in A. All LTTs entailed the incubation of 1.5×10^5^ PBMC in the presence of drug antigen for 5 days (37°C, 5% CO_2_) followed by pulsation with [^3^H] thymidine (0.5 µCi/well) and a further 16-hour incubation. Data presented as stimulation index of incorporated radioactivity relative to media controls (mean of triplicate cultures). (C) ICI therapy paradigm; the on-target effects of ICIs deregulate an individual’s tumor specific (efficacy) and autoreactive or otherwise deleterious (toxicity) T cells on a continuum. Respective thresholds may be set relative to each other in a given patient by the precursor tumor response and intrinsic susceptibility to autoimmune/aberrant T cell responses. BB, Bandrowski’s base; ICI, immune checkpoint inhibitor; PBMC, peripheral blood mononuclear cells; PPD; purified protein derivative; TT, tetanus toxoid.

Amidotrizoate responsive T cell clones were detected in the PBMC of this patient, which proliferated and secreted IFN-γ, granzyme B and perforin on challenge with amidotrizoate in a dose-dependent fashion (figure 1, E–J). *In-vitro* blockade of PDL-1 and CTLA-4 resulted in marked enhancement of memory responses to the antigen panel relative to the results obtained in the absence of the ICIs in 6/9 healthy donors (figure 2A). This was corroborated by a similar augmentation observed with PBMCs taken from 5/7 ICI patients prior to and after *in-vivo* treatment with ICIs (figure 2B).

## Discussion

Our case report highlights how the iodinated contrast medium amidotrizoate, which was previously well tolerated, led to SJS, a serious cutaneous adverse reaction, after the use of the ICI atezolizumab. Although causality was originally ascribed to atezolizumab, the positive rechallenge combined with mechanistic laboratory data implicated amidotrizoate. The use of atezolizumab thus appears to have shifted the immunological perception from tolerance/ignorance to a state of elicitation, resulting in SJS.

Our mechanistic data indicate the involvement of amidotrizoate-responsive T cells capable of secreting the cytolytic molecules granzyme B and perforin; both thought to be key mediators of SJS/TEN reactions.^7^ It is not clear whether the SJS that occurred in this patient was a result of enhanced priming responses of the naïve T cell compartment, as has been reported in previous studies.^2^ This is particularly unlikely for the initial, milder reaction, since the rapid onset of the reaction is not congruent with the time required for the initiation of a *de-novo* T cell priming response. Therefore, an alternative explanation is that a senescent memory component accrued through repeated historical exposure to amidotrizoate was invigorated by the use of atezolizumab. This is consistent with: (A) the known on-target pharmacologic effects of ICIs^8^ and (B) the enhanced recall lymphocyte proliferative responses observed in our study through *ex-vivo* and *in-vivo* administration of ICIs. The interaction we have described between atezolizumab and amidotrizoate may therefore represent a novel pharmacodynamic drug–drug interaction.

A clinically relevant ramification of our findings is that administration of ICI agents may inadvertently shift individuals towards an adverse immunological state in which hypersensitivity reactions are favored. While it is often reasonable to blame the ICI on its own for causing an irAE, and withdraw it, this may have negative consequences on survival from the malignancy. It is therefore also important to consider the role of concomitant therapies, particularly agents known to cause hypersensitivity reactions, in the manifestation of such reactions. The long half-life of the ICI antibodies^9 10^ raises the possibility that their effect on the immunological perception of compounds may persist for some time after administration, which is consistent with reports of various pharmaceuticals exhibiting poor tolerability when administered in sequence with ICIs.^4 5^

Our proof of concept study indicates utility of *ex-vivo* functional studies in investigation of deregulation imposed by ICIs within a given individual. An idealistic future paradigm as outlined in (figure 2C) stipulates that efficacy and toxicity may be on the same spectrum and that in theory patients could be prospectively stratified to treat only individuals who fall into a therapeutic window of immunological activity where tumor response is enhanced with an acceptable degree of IrAEs (figure 2C). While surrogate markers as offered herein do not encapsulate the relevant antigens with respect to efficacy and toxicity, they may reflect overall deregulation that ICIs would impose on an individual. *Ex-vivo* blockade in a functional T cell assay may therefore have potential as a novel avenue of exploration for prospective biomarkers of response. Future studies to this end should evaluate the corroboration of *ex-vivo* ICI blockade to *in-vivo* for various antigens, assess the transposition of this to tumor response and toxicity outcomes and attempt to categorize responses according to outcomes.

## References

[1] Metushi IG , Hayes MA , Uetrecht J . Treatment of PD-1(-/-) mice with amodiaquine and anti-CTLA4 leads to liver injury similar to idiosyncratic liver injury in patients. Hepatology 2015;61:1332–42. 10.1002/hep.27549 25283142

[2] Gibson A , Ogese M , Sullivan A , et al. Negative regulation by PD-L1 during drug-specific priming of IL-22-secreting T cells and the influence of PD-1 on effector T cell function. J Immunol 2014;192:2611–21. 10.4049/jimmunol.1302720 24510967 PMC3951492

[3] Kimura H , Hasegawa A , Takei I , et al. Characteristic pathological features of keratinocyte death in a case of Stevens–Johnson syndrome manifested by an immune checkpoint inhibitor. J Eur Acad Dermatol Venereol 2021;35. 10.1111/jdv.16872 32780890

[4] Ford M , Sahbudin I , Filer A , et al. High proportion of drug hypersensitivity reactions to sulfasalazine following its use in anti-PD-1-associated inflammatory arthritis. Rheumatology 2018;57:2244–6. 10.1093/rheumatology/key234 30107548

[5] Cui W , Cotter C , Sreter KB , et al. Case of fatal immune-related skin toxicity from sequential use of Osimertinib after pembrolizumab: lessons for drug sequencing in never-smoking non-small-cell lung cancer. JCO Oncol Pract 2020;16:OP2000489. 10.1200/OP.20.00489 32915710

[6] Pichler WJ , Tilch J . The lymphocyte transformation test in the diagnosis of drug hypersensitivity. Allergy 2004;59:809–20. 10.1111/j.1398-9995.2004.00547.x 15230812

[7] Peter JG , Lehloenya R , Dlamini S , et al. Severe delayed cutaneous and systemic reactions to drugs: a global perspective on the science and art of current practice. J Allergy Clin Immunol Pract 2017;5:547–63. 10.1016/j.jaip.2017.01.025 28483310 PMC5424615

[8] Wieder T , Eigentler T , Brenner E , et al. Immune checkpoint blockade therapy. J Allergy Clin Immunol 2018;142:1403–14. 10.1016/j.jaci.2018.02.042 29596939

[9] Mould DR , Meibohm B . Drug development of therapeutic monoclonal antibodies. BioDrugs 2016;30:275–93. 10.1007/s40259-016-0181-6 27342605

[10] Zhao L , Ren T-hua , Wang DD . Clinical pharmacology considerations in biologics development. Acta Pharmacol Sin 2012;33:1339–47. 10.1038/aps.2012.51 23001474 PMC4011353

